# Onsager-Casimir frustration from resistance anisotropy in graphene quantum Hall devices

**DOI:** 10.1103/physrevb.104.085418

**Published:** 2021

**Authors:** I-Fan Hu, Alireza R. Panna, Albert F. Rigosi, Mattias Kruskopf, Dinesh K. Patel, Chieh-I Liu, Dipanjan Saha, Shamith U. Payagala, David B. Newell, Dean G. Jarrett, Chi-Te Liang, Randolph E. Elmquist

**Affiliations:** 1 Physical Measurement Laboratory, National Institute of Standards and Technology (NIST), Gaithersburg, Maryland 20899, USA; 2 Department of Physics, National Taiwan University, Taipei 10617, Taiwan; 3 Physikalisch-Technische Bundesanstalt, Bundesallee 100, 38116 Braunschweig, Germany; 4 Department of Chemistry and Biochemistry, University of Maryland, College Park, Maryland 20742, USA

## Abstract

We report on nonreciprocity observations in several configurations of graphene-based quantum Hall devices. Two distinct measurement configurations were adopted to verify the universality of the observations (i.e., two-terminal arrays and four-terminal devices). Our findings determine the extent to which epitaxial graphene anisotropies contribute to the observed asymmetric Hall responses. The presence of backscattering induces a device-dependent asymmetry rendering the Onsager-Casimir relations limited in their capacity to describe the behavior of such devices, except in the low-field classical regime and the fully quantized Hall state. The improved understanding of this quantum electrical process broadly limits the applicability of the reciprocity principle in the presence of quantum phase transitions and for anisotropic two-dimensional materials.

## I. INTRODUCTION

It is widely known that graphene exhibits a variety of unique properties [1–4]. In certain forms, including epitaxial graphene (EG) grown on 4*H*-SiC, this versatile material has been identified as a practical way to develop resistance standards based on a robust quantum Hall effect (QHE) that appears over a useable range of magnetic fields (*B* fields), with the key feature being a well-quantized and extended resistance plateau [5–11]. Reported graphene-based standards operate almost exclusively at the filling factor *v* = 2, although a recent effort has been able to assess the viability of the *v* = 6 plateau [12]. For the *v* = 2 plateau, one expects the resistance value: $\frac{1}{2}\frac{h}{e^{2}}=\frac{1}{2}R_{\text{K}}\approx 12906.4037\Omega$, where *h* is the Planck constant, and *e* is the elementary charge.

In the past, the referenced graphene-based standards have been primarily single Hall bar devices, yielding a single operable value of resistance. However, recent advances in fabrication techniques have enabled the assembly of multiple Hall bars in parallel or in series to create resistance values of *qR*_K_, where *q* is a positive rational number [13–19]. Before these forms of standard devices are globally implemented, it is critical to disseminate best practices for characterization of the Hall resistance quantization for *B* field and current dependence. The symmetry of electrical conductance for opposite perpendicular directions of *B* field is one such criterion, as a result of the well-known Onsager-Casimir relations (OCRs) [20–22].

In this paper, we investigate the root cause of observed nonreciprocity in three types of large graphene quantum Hall devices: standard Hall bars with a length and width of 2 mm and 400 *μ*m, respectively, arrays of 13 parallel elements with quantized resistance *R*_K_/26 ≈ 992.8 Ω at the *v* = 2 plateau, and a 6.45 kΩ array consisting of eight elements in a series-parallel configuration. Electrical characterization of five Hall bars, four 13-element arrays, and one 8-element array yielded very similar results, and the data presented here are representative. All measurements were done in the four-terminal (4-T) measurement configuration, but the arrays are inherently two terminal (2-T) in their design, as required in precise QHE parallel array measurements [15]. Data were obtained by symmetrized lock-in measurements and with a direct current comparator (DCC) resistance bridge to assist in eliminating potential instrumental causes for observing nonreciprocity.

Our analysis determines that the structural anisotropies of EG contribute to the observed asymmetric Hall responses at intermediate magnetic fields. We posit that substrate morphology directly affects electron density variation that reduces the conductivity [23], and by extension, the anisotropic substrate morphology results in electrons experiencing nonuniform pseudomagnetic fields [24]. This results in backscattering, whose presence induces a device-dependent asymmetry, making the reciprocity relations limited in their capacity to describe the behavior of such devices.

## II. EXPERIMENTAL METHODS

### A. Sample preparation

EG films were grown on 4*H*-SiC substrates at temperatures near 1900 °C, with the sublimation of Si atoms allowing excess carbon at the surface to reorganize into a defect-free hexagonal lattice [25]. Chips were first diced from 4*H*-SiC(0001) wafers with atomically smooth Si-face surface obtained from CREE and chemically cleaned with a 5:1 diluted solution of hydrofluoric acid and deionized water. Before growth, some chips were coated with a very dilute solution of the carbon-based resist AZ 5214E in deionized water to utilize polymer-assisted sublimation growth (PASG) [26]. The silicon-face side of each chip was placed in close proximity (<2 *μ*m) with a polished glassy carbon slab (SPI Glas 22) to limit Si escape and improve graphene uniformity. The growth furnace was flushed with argon gas and filled to ~103 kPa from a 99.999% liquid argon source. The graphite-lined resistive-element furnace (Materials Research Furnaces Inc.) was held at 1900 °C for 4–5 min. The furnace heating and cooling rates were ~1.5 °C/s.

It should be noted that, after the growth, films were vetted by means of optical and confocal laser scanning microscopy to select those with monolayer coverage >99% (and uniform SiC step heights <1 nm), as described in a previous work [27]. For device fabrication, the EG layer was protected by a 20 nm layer of Pd/Au, followed by a photolithography process that defines the Hall bar and device contact pattern [28,29]. Thus, the Pd/Au layer and some exposed areas of SiC are covered with thicker Au to serve as the contact material with the device. For the 2-T array devices, a 100 nm layer of superconducting NbTiN was applied over the contacts to form device interconnects with superior performance [13]. The separation of the superconductor layer and the EG was >80 nm to prevent undesired quantum effects. Some of the chips were grown without PASG preprocessing, resulting in parallel SiC steps of increased height (1–5 nm) and >99% monolayer graphene, enabling us to quantify the influence of the steps themselves.

The final step for fabricating these quantum Hall devices was the functionalization process to regulate the electron density without the need for a top gate. The functional group used was Cr(CO)_3_, and it has been successfully implemented in a variety of other studies [30,31]. Hexahapto functionalization [(*η*^6^-graphene)-Cr(CO)_3_] was initiated with a small, nitrogen-filled furnace at 130 °C. The typical electron density of functionalized devices after being stored in air for at least 1 d is of the order of 10^10^ cm^−2^, and its uniformity varies on that same order across the entire chip [31], which can be compared with the typical values of inherent doping in EG of 10^13^ cm^−2^ [32]. As a control, some of the devices with the larger step edges were not functionalized to determine whether any sample anisotropies were attributable to the presence of Cr(CO)_3_.

**FIG. 1.**
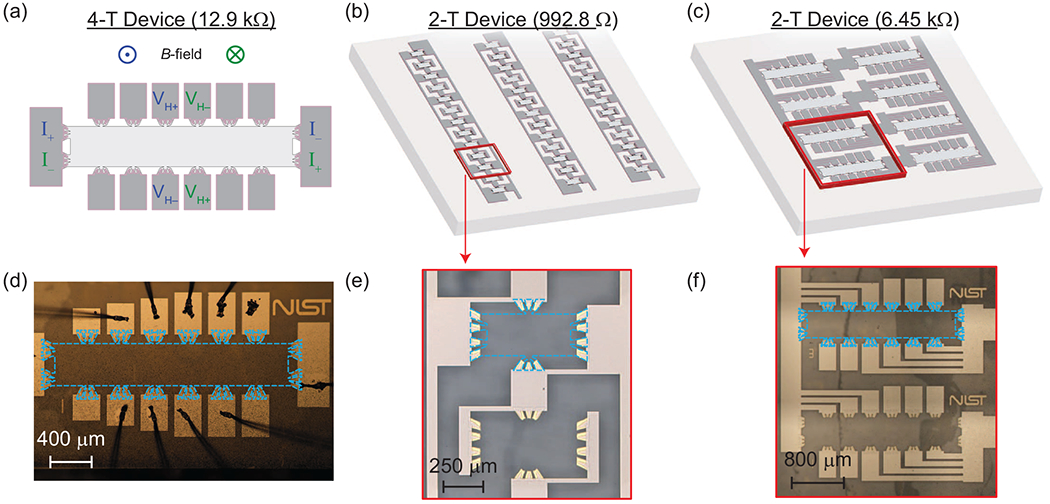
(a) An illustration of the 12.9 kΩ Hall bar devices. Two example four-terminal (4-T) measurements are shown and color-coded with the corresponding in- or out-of-page magnetic field direction. Illustrations of the (b) 992.8 Ω array and (c) 6.45 kΩ array devices are provided for clarity. Microphotographs of the respective postfabrication device elements are shown for the (d) 12.9 kΩ standard Hall bar, (e) 13-element array, and (f) 6.45 kΩ array.

A set of final device illustrations is shown in Figs. 1(a)–1(c), with corresponding images in Figs. 1(d) and 1(f). The first device type shown in (a) is a 12.9 kΩ standard Hall bar, suitable for 4-T measurements using distinct source-drain and voltage contacts. The second device type (b) is a 992.8 Ω array, composed of 13 Hall bars connected in parallel. The third device type (c) is a 6.45 kΩ array device composed of a 4 × 2 interconnected grid of Hall bars. Both array device types are exclusively 2-T but are measured as 4-T using separate voltage and current leads connected to the superconductor at the source and drain contacts, where we have implemented a multiple-branch design required to optimize the current flow and eliminate the effect of contact resistances [13,15]. The array designs also provide inherent reciprocity for reversal of the magnetic field direction in the QHE regime.

It should be noted that the main difference between the three kinds of devices is the contact configuration, e.g., only the single Hall bar devices could be measured using conventional 4-T magnetoresistance measurements at distinct contacts. In all array devices, elements share common electrical connections formed by the superconductor. For the 992.8 Ω array devices, symmetric sets of contacts access each Hall bar, whereas for the 6.45 kΩ array devices, sets of four and five contact pads contact each Hall bar element and are interconnected by NbTiN. With these differences, one can confirm that the measured anisotropies we will report are not the result of a particular contact configuration.

### B. Quantum Hall transport

**FIG. 2.**
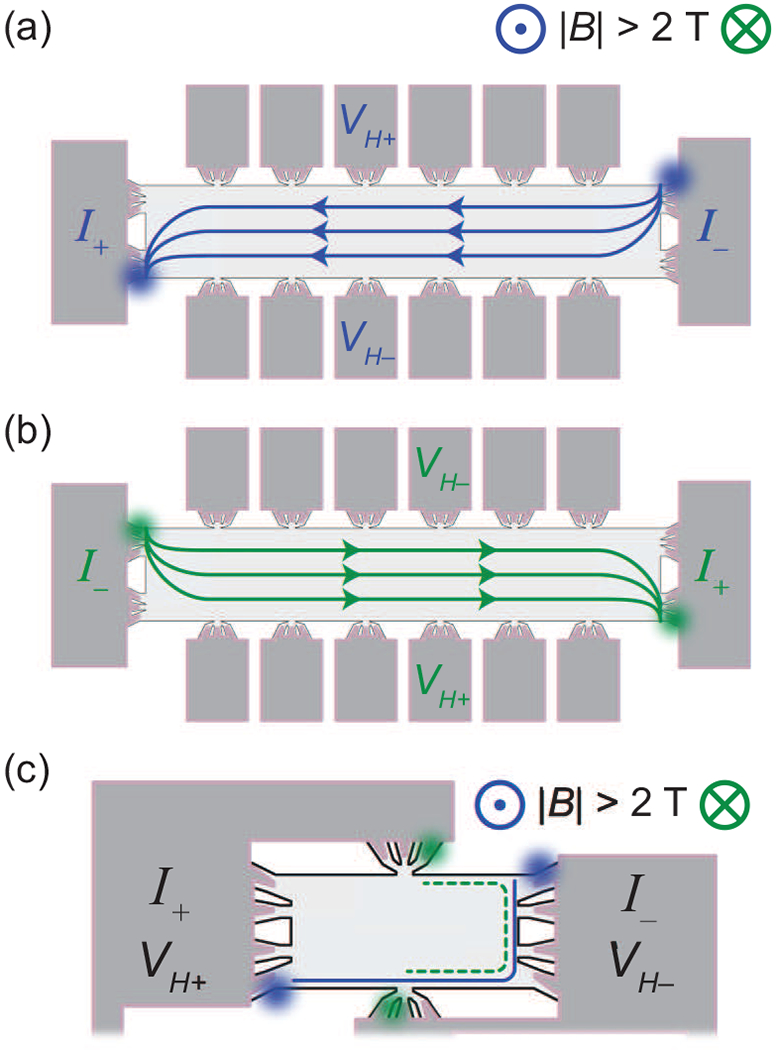
(a) and (b) Expected current behaviors for the standard Hall bar are illustrated and correspond to a four-terminal configuration for positive and negative *B* fields, respectively. (c) Example of an equipotential line in one element of an array device for positive (solid blue) and negative (dotted green) *B* field. Blurred circles represent hotspots or approximate points of electron entry or exit where most dissipation occurs in the quantum Hall effect (QHE) regime.

For quantum Hall transport measurements, a Janis Cryogenics ^4^He cryostat was used. The relevant data were collected at magnetic field values between 0 and ±9 T to characterize the magnetoresistances of the devices. Measurements were performed between 1.5 and 10 K with source-drain currents as high as 1 mA. Devices were annealed in vacuum, as described in Ref. [31], to obtain a desired electron density. The expected current behavior at low temperatures and varying magnetic fields are shown in Fig. 2. All blurred circles in Fig. 2 represent hotspots or areas associated with the majority of electron flow to and from the device electrical contacts in the QHE regime [33].

Regarding the 4-T and 2-T devices, we followed the symmetry relation described in Büttiker’s work [20]. Observations of strong asymmetry in resistance measurements for the 4-T measurements may appear as a result of local current flow behavior contributing to the measurement when the *B*-field direction is reversed without switching the voltage and current electrodes. Rather than measure the potentials purely associated with the reservoirs serving as the current source and drain, local potentials near the voltage terminals become embedded in the response [34]. Such local potentials may change when the *B*-field direction is reversed. For the 2-T array contact configurations, the same electrodes were used for both applying current and measuring voltage differences, and the current flow is derived from the normal and QHEs. The 2-T devices also do not suffer from resistance measurement errors due to low impedance lock-in amplifier inputs since any current drawn is supplied by the voltage or current source.

## III. OBSERVING NONRECIPROCITY

**FIG. 3.**
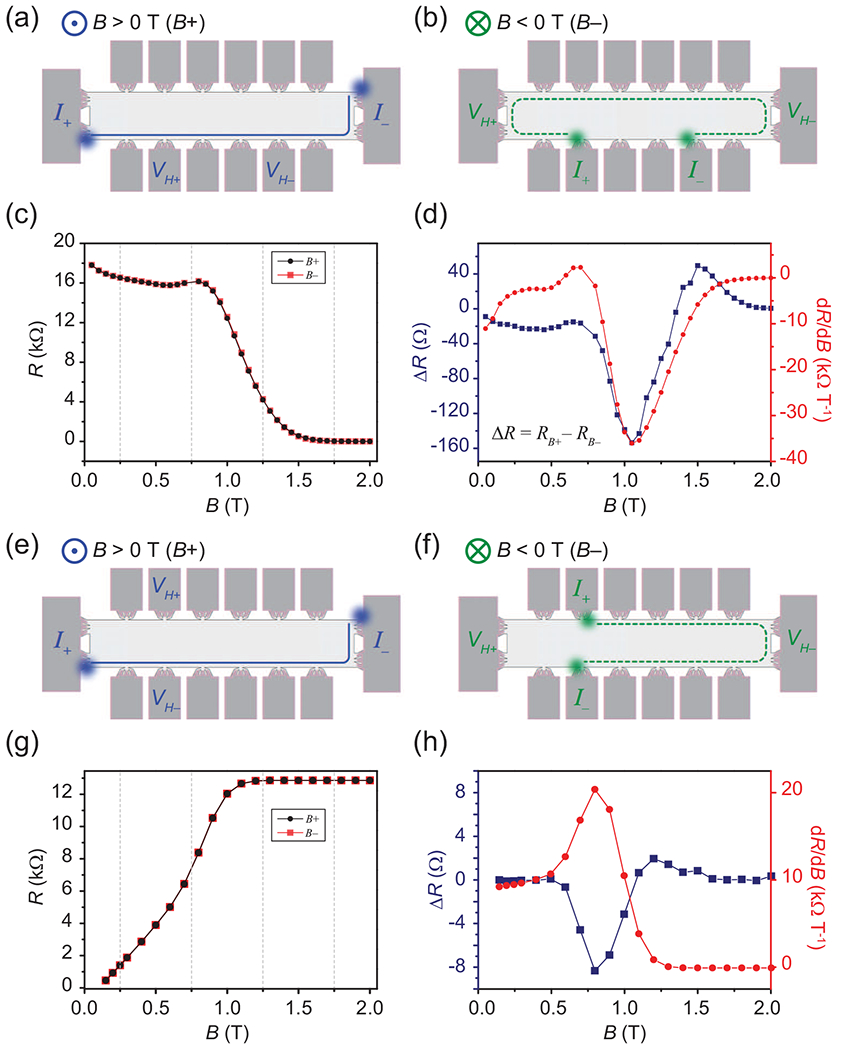
The differences in the measured longitudinal resistance [four terminal (4-T)] for (a) positive *B* field and (b) negative *B* field. Both illustrations have an example equipotential line as a reference. The illustrated measurement configurations correspond to the two sets of data in (c). By reflecting the negative *B*-field data about the vertical axis, nonreciprocity in the longitudinal resistances may be observed. (d) The difference between the two curves reveals a small yet reproducible effect. For comparison, the first derivative of the resistance with respect to the positive *B*-field case is shown in red. (e)–(h) The same analyses were conducted for the corresponding Hall resistances. Note that the epitaxial graphene (EG) on this device was grown via the polymer-assisted sublimation growth (PASG) method, which greatly reduces any inhomogeneity or anisotropy due the step edges. These devices were functionalized with Cr(CO)_3_.

One electrical measurement configuration is equated to a second one by means of the OCR [20–22], wherein the current terminals are exchanged with the voltage terminals and the positive current probe becomes the positive voltage probe, and likewise for the negative terminals. Illustrations are shown in Fig. 3 for (a) positive *B* fields and (b) negative *B* fields. For this first set of measurements, the focus is on the single-device longitudinal resistance (4-T), whose corresponding data are in Fig. 3(c). To compare the resistances from the positive and negative *B*-field cases, the latter is reflected about the vertical axis, identical to taking the absolute value of the magnetic field reading.

Effects of hysteresis due to trapped flux in the superconducting magnet were minimized by the experimental procedure. For the 4-T measurements, fixed *B*-field values were used rather than continuously ramping the field. The *B* field was adjusted to the desired value, always with increasing magnitude of *B*, followed by resistance measurements using a fixed driving current. All first-order thermoelectric effects were removed by averaging the measured resistance values for positive and negative current directions.

Upon first glance, the longitudinal resistances overlap, but upon taking the difference of the two curves, as shown in Fig. 3(d), a small yet measurable and reproducible change is visible (blue curve, left axis). The global minimum of this curve aligns well with the global extremum of the first derivative of the resistance with respect to the positive *B*-field case (red curve, right axis). In Figs. 3(e) and 3(f), illustrations of the 4-T Hall measurements are shown for the positive and negative *B*-field cases, respectively. The same operations were conducted for the corresponding Hall resistances, as seen in Fig. 3(g). The resistance difference, defined as Δ*R* = *R_B+_* − *R_B−_*, in Fig. 3(h) is more than an order of magnitude smaller than the longitudinal case, and although the resistance derivative is of similar order, the sign is reversed.

**FIG. 4.**
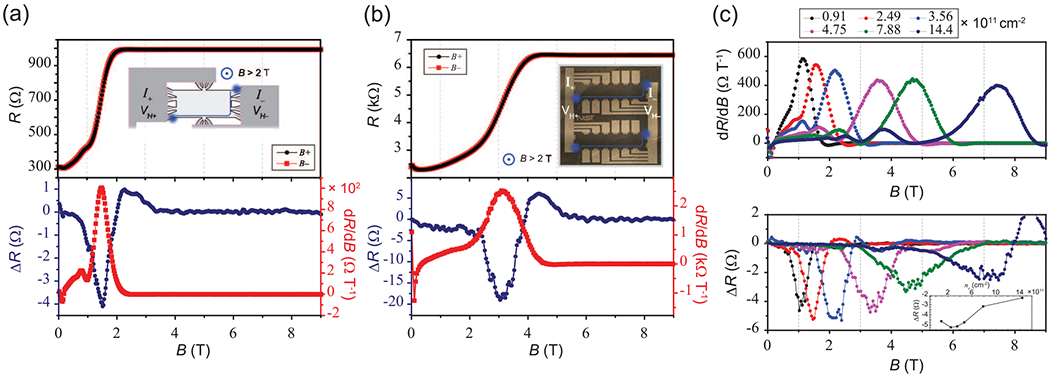
Measurements using a two-terminal (2-T) device in a four-terminal (4-T) measurement configuration for the (a) 992.8 Ω array and (b) 6.45 kΩ array devices (insets show example equipotential line in solid blue). The top panels show the combined Hall and longitudinal resistance, as well as the corresponding positive *B*-field measurement configuration, and the bottom panels show Δ*R* in blue and first derivative of the positive *B*-field case in red. (c) Both the derivative of the combined resistance curve for the 992.8 Ω array (positive *B*-field case) and Δ*R* between the two conditions are shown as a function of electron density. In the ideal case, the bottom panel should yield no differences for devices obeying the Onsager-Casimir relation (OCR).

The observations of nonreciprocity are not exclusive to 4-T devices. Using 2-T 992.8 Ω and 6.45 kΩ array devices, similar differences in the combined (Hall and longitudinal) resistances can be seen. In Figs. 4(a) and 4(b), the mixed resistance (top panel) response maintains a symmetric appearance but yields a Δ*R* and first derivative behavior (blue and red curves in the bottom panel, respectively) like the 4-T configuration. In Fig. 4(c), the derivative of the 2-T device resistance curve (positive *B*-field case) and Δ*R* are shown as a function of magnetic field for different electron densities. In the ideal case, the bottom panel of Fig. 4(c) should yield no differences for devices obeying the OCR. The inset of the bottom panel shows the peak value of Δ*R* as a function of electron density and suggests that the nonreciprocity gradually decreases with higher *n*.

## IV. DETERMINING THE CAUSE OF NONRECIPROCITY

### A. Device inhomogeneities

The aforementioned observations of nonreciprocity are consistent within all three device types, where multiple devices were measured within each category, prompting a more careful analysis. More data are available in the Supplemental Material [35], including comparisons of the homogeneity of the electron density in all device types as well as the nonreciprocity behavior as a function of injected current. Additionally, consistent nonreciprocity observations for the *ν* = 6 plateau are provided.

**FIG. 5.**
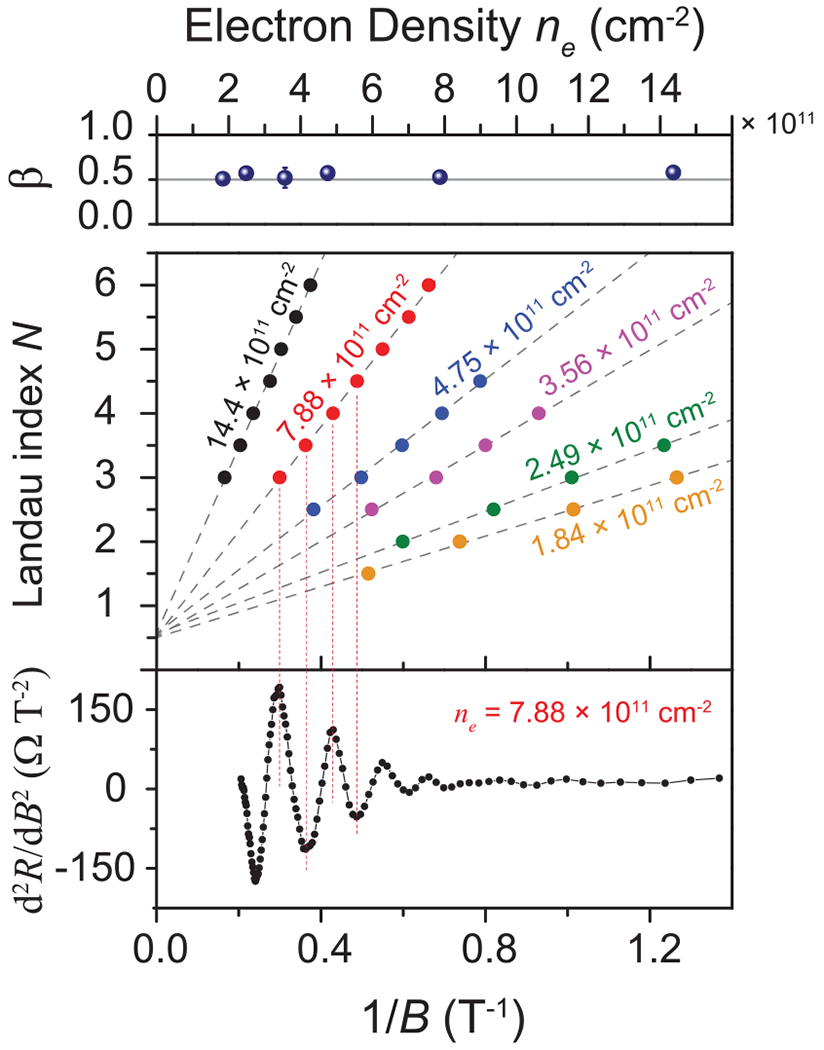
Data are shown for the 992.8 Ω array. The fraction in the top panel *β* is the *y* intercept for the lines in the middle panel. The middle panel shows the Landau index *N* plotted against 1/*B* for a set of six widely spaced electron densities. The bottom panel shows the second derivative of one of the electron density cases, and its regular periodicity confirms sample homogeneity. This behavior is universal to all tested devices. Error bars indicate a 1σ uncertainty of the data collected at the corresponding point.

One immediate consideration to make when seeing any data that do not conform exactly to well-established principles is the quality of the device. Assuming a uniform electron density [35], one may confirm the quality of the device by inspecting the transport characteristics. In Fig. 5, three such checks are presented. First, the fraction in the top panel *β* is the *y*-axis intercept for the lines in the middle panel. A value of *β* = 0.5 implies that the behavior of the carriers inside the graphene are Dirac-fermionlike. It also affirms that the Cr(CO)_3_ functionalization did not influence the behavior of those carriers. Furthermore, it supports the notion that the EG is of high quality since the results are like those from other works utilizing high-quality graphene [36]. Note that the error bars are smaller than the data points.

The middle panel of Fig. 5 shows the Landau index *N* is plotted against the inverse of the applied *B* field for the same set of electron densities as seen in Fig. 4, and the usual linear relationships between these two quantities were verified. Note that the positions of the Landau indices are obtained from the second derivative of the measured resistance [37]. The bottom panel of Fig. 5 shows the second derivative of one of the electron density cases, and its periodicity serves as another confirmation of sample homogeneity (see Supplemental Material [35]).

**FIG. 6.**
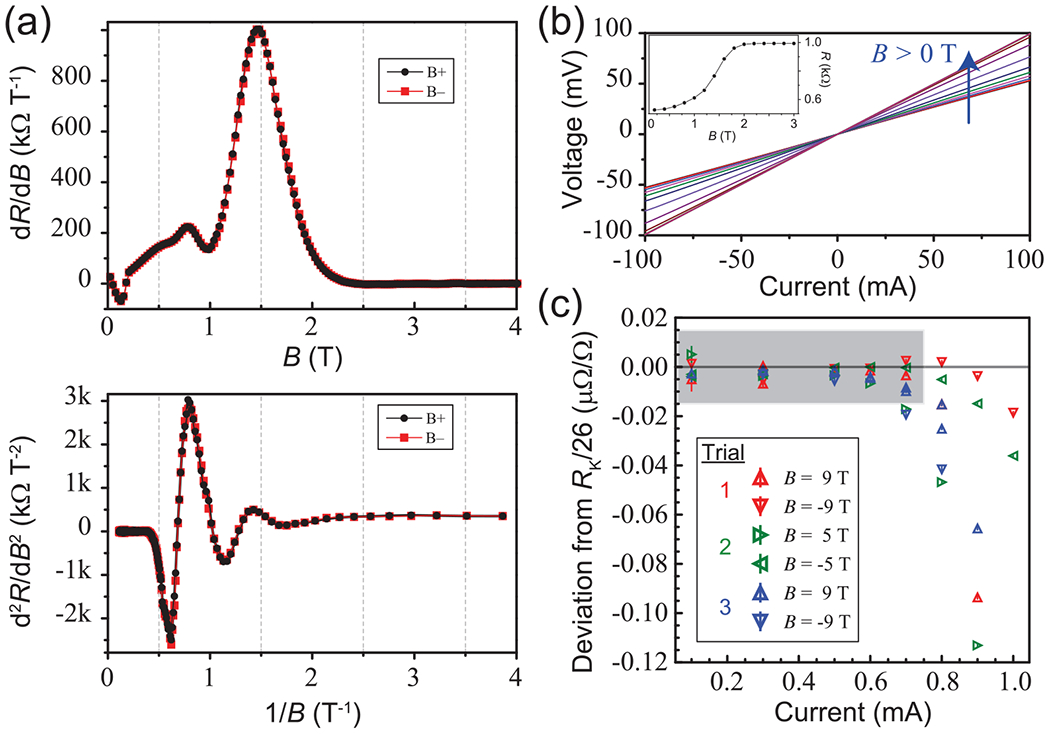
(a) The first and second derivatives of both resistance measurements for the 992.8 Ω array device are shown in the top and bottom panels, respectively. The similarity between *B*+ and *B*− confirms that the device inhomogeneity, averaged over the 13 elements, does not determine Δ*R* and suggests that the device quality is not a factor in why those differences appear. (b) Zero-field and low-field *I-V* curves are measured at 1.6 K to verify device linearity, another indicator of homogeneity. (c) Direct current comparator (DCC) measurements verify that, in the high-field limit, the resistance for both *B*-field directions approaches the value $\frac{R_{\text{K}}}{26}\approx 992.8$ Ω to better than one part in 10^8^ for currents up to 700 *μ*A, confirming that this quantum Hall effect (QHE) array device utilizes highly homogeneous graphene.

Additional methods were utilized to evaluate the possible contributions of monolayer EG quality and device contact arrangements to nonreciprocity. In Fig. 6(a), the first and second derivatives of 2-T device measurements for both magnetic field polarities are shown for the 992.8 Ω array device in the top and bottom panels, respectively. The similar appearance confirms the symmetry exhibited by the device as it transitions to the quantum Hall regime. Despite this symmetry, the Δ*R* behavior still showed peaks aligned with the first derivatives, suggesting that general device quality is not a major contributor in Δ*R*.

As shown in Fig. 6(b), both the zero-field and low-field *I-V* curves are measured at 1.6 K to verify device linearity, which is another indicator of general quality and homogeneity. The fact that our devices are electrically linear validates the basic requirements for reversed-field reciprocity [21]. The final device quality check was performed with a DCC. These precision measurements [Fig. 6(c)] show, in the high-field limit of 5 to 9 T, that data from both *B*-field polarities approach the fully quantized state for currents <~ 700 *μ*A. This behavior seen with the DCC confirms that the 992.8 Ω array device was fabricated from the highest quality film growths, as were all devices.

### B. Anisotropy in EG

**FIG. 7.**
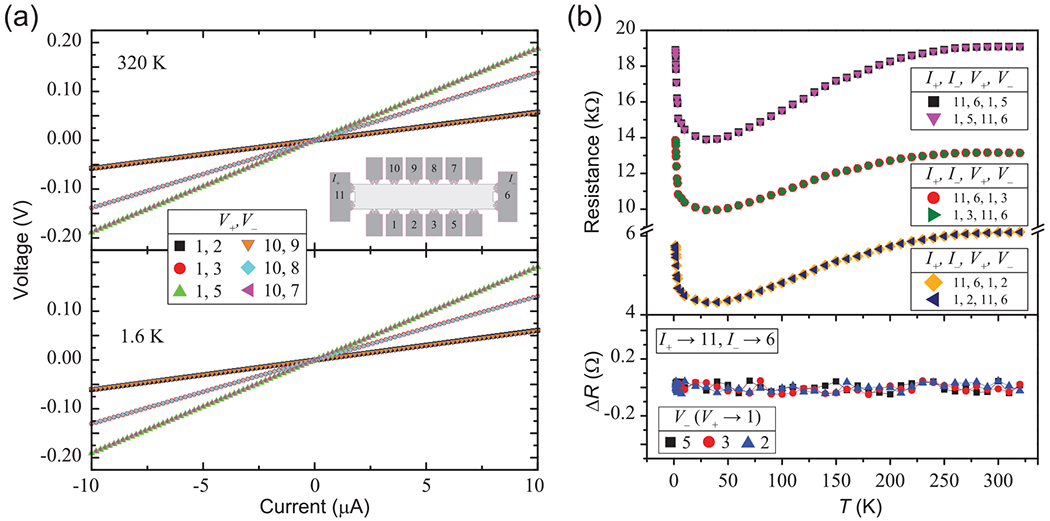
(a) The *I-V* curves at zero field are shown for an example device at 320 and 1.6 K, in the top and bottom panel, respectively. The linearity supports overall device homogeneity. (b) Temperature-dependent measurements of the Onsager-Casimir relation (OCR) at zero field demonstrate that the presence of a *B* field gives rise to the observed Δ*R* in other data.

The data shown in Fig. 4 are consistent with all devices, namely, that all Δ*R* become small at low *B*, in the linear Hall regime. To explore this behavior more closely, zero-field measurements were first conducted to confirm whether we can measure the OCR accurately (to within the noise of the equipment). Without any applied *B* fields, the OCR is expected to hold. Data supporting this zero-field expectation are shown in Fig. 7. Not only do the devices demonstrate linearity at high temperatures, but one can also see that Δ*R*, as a function of temperature, remains at zero within the equipment noise. This observation supports the notion that device quality is not a significant contributor to observed asymmetries. We therefore conclude that the cause of the OCR asymmetries in our measurements is related to *B*-field-induced asymmetry.

**FIG. 8.**
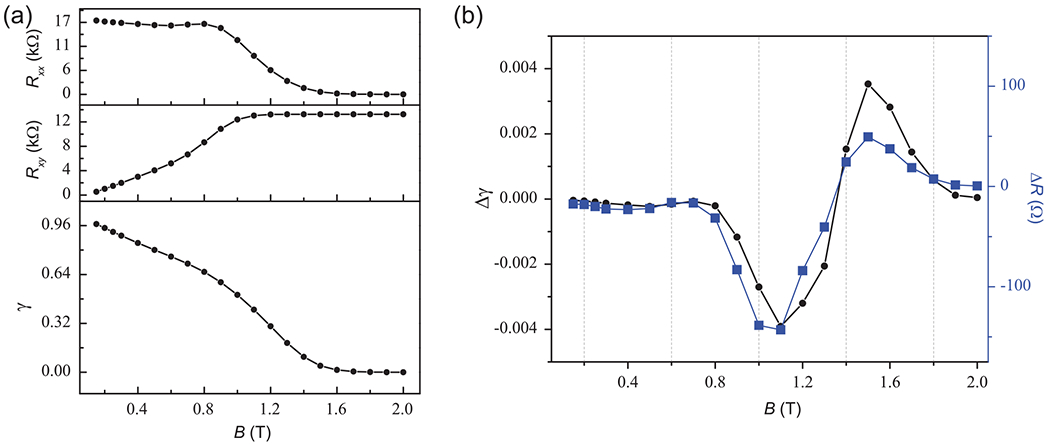
(a) An example set of longitudinal and Hall resistance measurements (top and middle panel, respectively) are shown to compare with the calculated backscattering strength as a function of *B* field (bottom panel) for a 12.9 kΩ device. (b) The difference of the backscattering strength parameters is shown as a function of *B* field (black circles, left vertical axis), with the corresponding observed Δ*R* shown as blue squares (right vertical axis).

In micrometer-scale EG-based quantum Hall devices, it has been reported that *B*-field asymmetry mainly resulted from electron backscattering and was a gate-tunable phenomenon [38]. Therefore, the OCR may not hold if backscattering takes place in our system. To further support the notion that Δ*R* results from backscattering, the backscattering strength was calculated for the injected current in the longitudinal direction using the following formula: $\gamma =\frac{R_{L}}{R_{L}+R_{H}}$ [39]. Figure 8(a) shows the backscattering strength as a function of positive *B* field, along with the corresponding *R*_*xx*_ and *R*_*xy*_. In Fig. 8(b), the difference of backscattering strength Δ*γ* = *γ*_*B*+_ − *γ*_*B*−_ for low fields shows a strong similarity to the measured Δ*R*.

It should be noted that the backscattering strength shown in Fig. 8(a) combines all sources of backscattering, including those from differences in the population of Landau levels as the *B* field changes. This population change has been reported as being inherently linked to *B*-field symmetry [21]. Therefore, the observed backscattering-related *B*-field asymmetry in our devices must originate by some other cause.

In the case of EG, the presence of SiC step edges precludes the uniform distribution of electrons over the device area. It can thus be stated that the electron density is directly influenced by local substrate morphologies, which in turn result in nonuniform *B* fields acting on electrons in any deformed areas [40–42]. The strength of any backscattering depends on the applied *B* field [43,44]. At low *B* fields, a diverse population of states exists in the device due to the transitions between neighboring Landau levels, electron density fluctuations, and nonuniform *B* field. Thus, electronic states are readily available for backscattering events. This increased backscattering results in a greater *B*-field asymmetry, thus intensifying the breakdown of the reliability of measuring the OCR.

At high *B* fields, this diverse population no longer propagates as such through the device due to the large spacing between the zeroth and first Landau levels in EG. The Fermi level, though susceptible to perturbations, still remains within the Landau gap. Therefore, even if the electron density is not uniform, wide separation of Landau levels is expected to suppress any backscattering. This phenomenon is unique in EG-based systems because the *ν* = 2 plateau persists for large *B* fields [33]. Note that the backscattering from the step edges is *B*-field asymmetric since the current path changes with the direction of the *B* field (Fig. 3). The morphology and number of the step edges are different for each current path, thus resulting in different backscattering strengths.

Because these devices are macroscopic (surface dimensions >100 *μ*m), any asymmetric contributions to the resistance from backscattering are averaged over many random current paths. Our data show that anisotropy is inherently a phenomenon observable during the phase transitions of the quantum Hall states. In smaller-scale devices, anisotropies are expected to have larger impacts [21,26,45–48], with higher temperatures also causing a suppression of backscattering (for temperature suppression data, please see the Supplemental Material [35]). For this paper, since EG was grown with different methods, different surface morphologies were accessible. Furthermore, EG properties varied in that some devices were functionalized, and some were not, and different contact configurations and contact pad compositions were used, while OCR asymmetries were consistently observed.

**FIG. 9.**
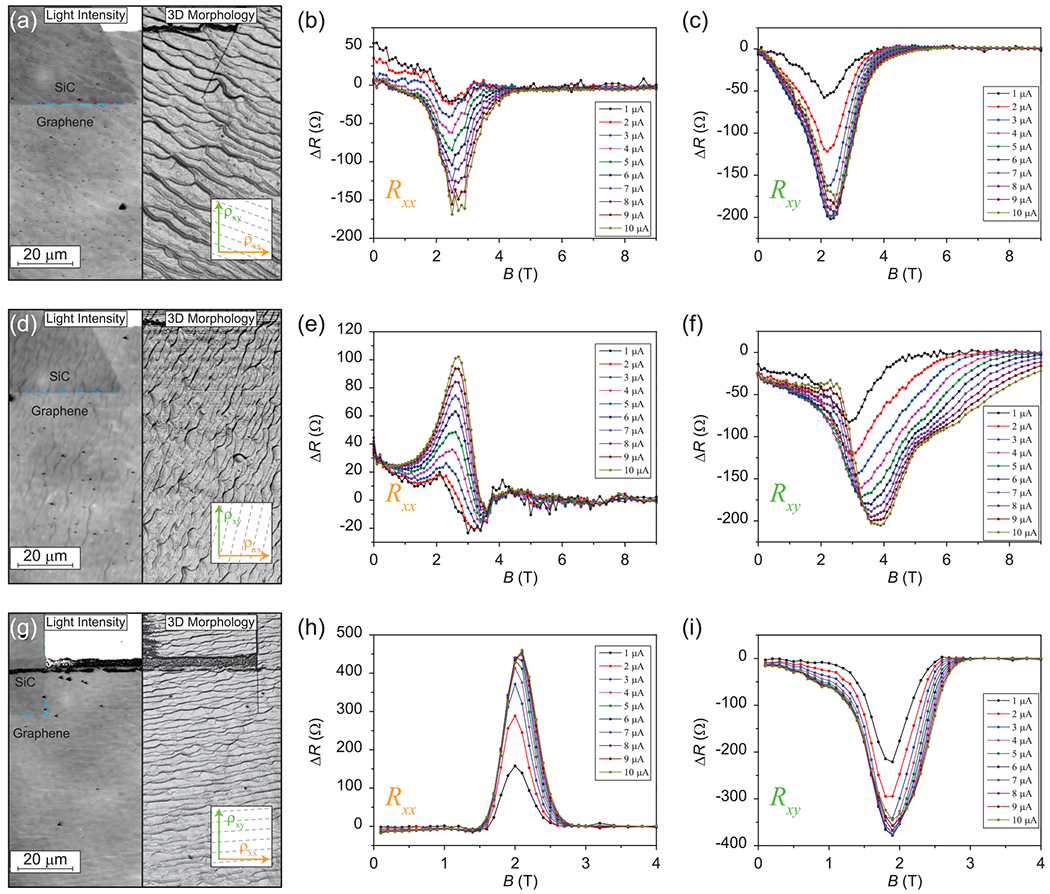
(a), (d), and (g) Confocal laser scanning microscopy images of three orientations of step edge on three single epitaxial graphene (EG) devices are shown, with an approximate artistic rendering of the directionality of the step edges shown in the lower right inset. The left and right halves of each panel show the light intensity and three-dimensional morphology images, respectively. (b), (e), (h) Differences of the longitudinal resistances are shown as a function of perpendicular magnetic field at different current levels, with variable magnitudes and sign for the step orientations depicted to their left. (c), (f), (i) Similar data are shown, but instead for the Hall resistances. Δ*R* increases in magnitude in all cases as the injection current is increased; however, this effect saturates at higher current for some cases, as shown in (c), (f), (h), and (i).

Due to the complexity and size of the EG system, as well as the subtle, sample-dependent differences in how the step edges form, simulations or comparisons of the absolute values of resistances may not be feasible approaches for assessing anisotropies. However, because one may assume that most backscattering takes place at the SiC steps, one can expect an influence from the step edge orientation on the measured anisotropy. Three single Hall bar devices are shown in Figs. 9(a), 9(d), and 9(g), with different step orientations (with orientations illustrated in the lower right insets). Differences for the longitudinal resistances are shown in Figs. 9(b), 9(e), and 9(h) as a function of *B* field for varying current levels, with the corresponding Hall Δ*R* shown in Figs. 9(c), 9(f), and 9(i).

Backscattering anisotropy appears to increase as Hall quantization develops, implying that the direction of the current (and its angle to the step edges) plays a significant role in the measured OCR asymmetries. For instance, by looking at the step edges in Fig. 9(a) (nearly 45°), Δ*R* for *R*_*xx*_ and *R*_*xy*_ have a similar response with *B* field (the incident angle for *B*+ and *B*− is nearly identical). Furthermore, when different sets of contact pads were used, there was a small impact on this relationship, and the reciprocity differences for *R_xx_* and *R_xy_* were nearly identical (within 10% of one another).

In Figs. 9(d) and 9(g), the step edges are nearly perpendicular or parallel, respectively, to the long axis of the devices, and Δ*R* for *R*_*xx*_ and *R*_*xy*_ have completely different characteristics when compared with one another. The most obvious divergence is in the signs, where the perpendicular step edge case has both maxima and minima in the field dependence. Since the backscattering process is not the same when the electrons sample other regions, comparing data for other contact pads was not as fruitful as for the first case. Regardless, it remains evident that *R*_*xx*_ and *R*_*xy*_ are sensitive to the step edge orientation [40]. Furthermore, Δ*R* undergoes drastic increases with rising current, particularly for *R_xy_*, but this begins to saturate at relatively low current level, as seen in other related work [45].

Given that our observations and parametric tests on these devices are yielding consistent results with other work, our results suggest that the use of the OCR is not always a reliable guide to the quality of electrically linear systems and EG devices in particular. For devices that have inherent surface morphology differences or anisotropic electrical properties, additional or alternative tests are warranted.

## V. CONCLUSIONS

In this paper, we determined a probable cause of OCR nonreciprocity in three types of quantum Hall devices. After verifying the functionality of each device and eliminating many possible sources of asymmetry, it is confirmed that substrate-induced morphology directly affects the current flow by inducing electron density variation and, by extension, results in electrons experiencing nonuniform magnetic fields.

This leads to backscattering, whose presence ultimately induces a device-dependent asymmetry in the quantum Hall transitions. This asymmetry renders the OCR limited in their capacity to accurately characterize the Hall and longitudinal resistances of these devices. Therefore, these observations may be useful in any experiment relying on the broader Onsager relations because careful assessment of the current flow is required.
